# Individually or as a Team—The Immunological Milieu in the Lung Caused by Migrating Single-Sex or Mixed-Sex Larvae of *Schistosoma mansoni*

**DOI:** 10.3390/pathogens12121432

**Published:** 2023-12-08

**Authors:** Miriam Bischofsberger, Cindy Reinholdt, Tim Alexander Dannenhaus, Johann Aleith, Wendy Bergmann-Ewert, Brigitte Müller-Hilke, Micha Löbermann, Emil C. Reisinger, Martina Sombetzki

**Affiliations:** 1 Division of Tropical Medicine and Infectious Diseases, Center of Internal Medicine II, Rostock University Medical Center, Ernst-Heydemann-Straße 6, 18057 Rostock, Germany; miriam.bischofsberger@uni-rostock.de (M.B.); cindy.reinholdt2@uni-rostock.de (C.R.); tim.dannenhaus@uni-rostock.de (T.A.D.); micha.loebermann@med.uni-rostock.de (M.L.); emil.reisinger@uni-rostock.de (E.C.R.); 2 Core Facility for Cell Sorting and Cell Analysis, Rostock University Medical Center, 18057 Rostock, Germany; johann.aleith@med.uni-rostock.de (J.A.); brigitte.mueller-hilke@med.uni-rostock.de (B.M.-H.)

**Keywords:** *Schistosoma mansoni* infection, lung stage larvae, single-sex infection, immune milieu, lung inflammation

## Abstract

While the lung is considered an efficient site for stopping the larvae of the acute *Schistosoma* spp. infection phase from migrating through extensive inflammatory responses in the surrounding tissues, little is known about these processes. To date, the highest resistance to infection has been achieved in experimental studies with radiation-attenuated cercariae immunization, which elicits a strong Th1/Th2 response in the lung and results in up to 80% protection. Based on our own studies demonstrating a systemic, unpolarized Th1/Th2 response resulting from infection with male or female *Schistosoma mansoni*, we hypothesize that this atypical immune response is already detectable during the pulmonary passage of parasite larvae. Therefore, we examined the immune milieu in the lungs of mice caused by migrating schistosome larvae, either male or female (single-sex groups) or male + female (bisexual control), 4 and 16 days after infection in bronchoalveolar lavage and lung tissue by flow cytometry, qPCR, and multiplex analyzes. Our results show only minor differences in the inflammatory profile between the single-sex groups but significant differences compared with the bisexual control group. Both single-sex infected groups have increased expression of inflammatory markers in lung tissue, higher numbers of cytotoxic T cells (day 4 post-infection) and more T helper cells (day 16 post-infection), compared with the bisexual control group. A single-sex infection, regardless of whether it is an infection with male or female cercariae, causes an immune milieu in the lung that is clearly different from an infection with both sexes. In terms of identifying therapeutic targets to achieve resistance to re-infection, it is of great scientific interest to identify the differences in the inflammatory potential of male or female and male + female parasites.

## 1. Introduction

In the 21st century, schistosomiasis remains a widespread and devastating tropical disease, accounting for an estimated 1.9 million disability-adjusted life years (DALYs) globally [1]. The pathogens are trematodes (known as blood flukes) of the genus *Schistosoma* spp. The disease is characterized by the host’s immune response to schistosome eggs entrapped in tissues, leading to granulomatous inflammation and fibrosis [2,3,4]. In 2018, the World Health Organization (WHO) reported nearly 240 million cases of *Schistosoma* spp. infections, primarily in Africa (with a focus on South Africa), the Middle East, and South America [5]. Most of the affected regions are among the poorest countries in the world and face difficulties in providing basic health care at the primary level. However, there has been growing concern about confirmed cases of schistosomiasis in southern Europe, specifically Corsica and Spain [6,7]. These cases underscore the emerging public health threat even in Western industrialized nations due to climate change and human migration [8].

Despite great progress towards a vaccine, no candidate has yet shown satisfactory efficacy in clinical trials [9,10]. It has been shown that even a reduction in infection load would be sufficient to prevent a severe course of the disease [11]. To our knowledge, the most successful experimental immunization so far has been achieved with radiation-attenuated (RA) cercariae together with the Th1 cytokine IL-12 as adjuvant, which elicits a strong Th1 response and up to 80% protection [12].

The reduced migration speed of the radiation-treated cercariae combined with an augmentation of the immunogenicity of the larval stage allows the host to develop a more robust immune response. This response weakens newly arriving larvae and also maintains inflammation in the lungs for a period of time, providing protection against secondary infections. Passage through the lungs, whether via lung tissue or the vascular system, is an essential part of host migration and maturation for many parasitic worms. Helminths such as *Schistosoma* spp. can cause significant mechanical and chemical damage to the pulmonary vasculature and elicit a distinct type 2 immune response characterized by eosinophilia, goblet cell proliferation, increased mucus production, and cough and wheeze [13,14,15].

It has been assumed for a long time that mainly the eggs are responsible for the development of a host immune response [16,17]. However, studies using unisexual infection models have clearly shown that the worms themselves can effectively modulate the immune system [18,19,20]. In addition, male and female schistosomes have different immunomodulatory effects on the host. As early as 1953, experiments with rhesus monkeys showed that pre-infection with male schistosomes can induce immunity against subsequent bisexual infections [21]. Later studies confirmed that the observed protective effect was indeed dependent on the sex of the parasite [22]. Parallel findings in mouse models have revealed sex-specific differences in the triggered immune response. In particular, male worms were found to elicit a more robust immune response than females in single-sex infections [20].

In our own work, we have shown that in single-sex infection of mice over 52 weeks, both worm sexes are strongly immunogenic and induce organ-damaging inflammatory responses. Using transcriptome analyses on spleen samples from single- or bisexually infected mice, we demonstrated a clear clustering of regulated genes in the single- or bisexually infected experimental groups. Furthermore, we found that adult schistosomes elicit a non-polarized Th1/Th2 immune response in the host independently of the eggs [23,24].

In addition to the high immunogenic potential of both worm sexes, we examined the effect of single-sex infection on re-infection. We demonstrated differences in the inflammatory processes triggered by male or female schistosomes. While initial infection with female *Schistosoma* (*S.*) *mansoni* cercariae resulted in a reduced Th2 response upon subsequent bisexual infection, as evidenced by smaller liver granulomas and less pronounced liver fibrosis in these mice, male *S. mansoni* cercariae triggered a strong innate immune response that resulted in a visible reduction in worm and egg burden in the livers of these infected mice upon bisexual re-infection [25,26].

Since the lungs represent an important stage in the development of the parasites, we assume that the lung stage larvae (LSL) sex plays an important role in the inflammatory response necessary for elimination. We address these hypotheses in this study by infecting mice with male or female and male and female cercariae and imaging an immune profile of inflammation at two time points during lung passage.

## 2. Materials and Methods

### 2.1. Ethics

All experiments involving the infection of mice were conducted in strict adherence to the regulatory guidelines set forth by the German Society for Laboratory Animal Science and the European Health Law of the Federation of Laboratory Animal Science Associations. The animal experimentation protocols underwent rigorous review and received approval from the Mecklenburg-Vorpommern State Office for Agriculture, Food Safety, and Fisheries (TVA 7221.3-1-70/21). Stringent measures were implemented to mitigate and minimize any potential suffering experienced by the animals.

### 2.2. Schistosoma mansoni Infection of Mice

The life cycle of *Schistosoma mansoni*, particularly the Belo Horizonte strain, was maintained by using *Biomphalaria glabrata* (*B. glabrata*) freshwater snails as intermediate hosts and 6–8-week-old female NMRI mice as definitive hosts. To obtain cercariae of exclusively male or female origin for subsequent infection of mice, *B. glabrata* snails were exposed to a single Schistosoma mansoni miracidium. The sex of *S. mansoni* is already determined in the eggs [27], hence exposure to a single miracidium leads to a single-sex infected snail. Following a six-week infection period, individual snails were subjected to a robust light stimulus to induce the production of cercariae. Subsequently, the sex determination of cercariae was conducted through end-point polymerase chain reaction (PCR), employing primers specific to the female sex, as detailed in prior studies [25]. The mouse infections were performed percutaneously using the water bath method. In this process, the mice are infected using a defined number of cercariae in 50 mL of water for 2 h. For determination of the larvae burden peak in the lungs, C57BL/6j mice were infected with 300 mixed cercariae for 5, 6, 7, 8, 10, and 14 days (every group included *n* = 5 mice) (Supplementary Figure S1). To investigate the immune milieu during larvae migration through the lungs, C57BL/6j mice were infected with 100 either male, female, or mixed-sex cercariae over 4 and 16 days (*n* = 7, respectively), an early time point before peak and a late time point after the peak of 6 days post-infection. Uninfected mice served as healthy control group (*n* = 6). The mice were euthanized by final blood collection under ketamine/xylazine anesthesia. After blood collection, a bronchoalveolar lavage (BAL) was performed, and the lungs were collected for further analysis.

### 2.3. Quantitative Real-Time PCR Analysis

Total RNA was extracted from snap frozen lung samples using the RNeasy Plus Mini Kit (Qiagen, Hilden, Germany). The RNA concentration was determined using the Colibri microvolume spectrometer (TiterTek-Berthold, Bad Wildbad, Germany). Subsequently, 500 ng of total RNA was subjected to reverse transcription using the High-Capacity cDNA Reverse Transcriptase Kit (ThermoFisher, Erlangen, Germany) according to the manufacturer’s instructions to synthesize cDNA. Each quantitative real-time polymerase chain reaction (qRT-PCR) was performed using 2 µL of cDNA in a final reaction volume of 10 µL. All samples were run in triplicate with a standard deviation of <0.25. RT-PCR was performed using the following TaqMan Gene Expression Assays for pro-inflammatory genes: *tnf-α* Mm00443258_m1, *icam-1* Mm00516023_m1, *ifn-γ* Mm01168134_m1, *il-13* Mm00434204_m1, and anti-inflammatory genes: *foxp3* Mm00475162_m1, *pd-l1* Mm03048248_m1, *hmox* Mm00516005_m1. The amplification cycles were executed on the QuantStudio 3 instrument (Thermo Fisher Scientific, Dreieich, Germany) with the following reaction conditions: an initial step at 50 °C for 2 min, followed by 10 min at 95 °C, then 45 cycles at 95 °C for 5 s, and finally at 60 °C for 1 min. Relative quantification was performed using the ΔΔCt method. Gene expression values were normalized to the endogenous reference gene *gapdh*, employing the Rodent GAPDH control reagent from Thermo Fisher Scientific, Germany, and presented as normalized expression values relative to the naive controls.

### 2.4. Flow Cytometric Analysis

In order to analyze bronchoalveolar lavage cells, 500 µL of ice-cold PBS was slowly injected into the lungs through a small incision between the cartilages of the trachea twice and aspirated again. After centrifugation, the supernatant was frozen and stored at −80 °C for further use. Single-cell suspensions from lung tissue were prepared from a defined part of the lung that had been digested beforehand. The tissue was minced and incubated in 3 mL RPMI Medium (RPMI 1640 containing 100 U/mL penicillin and 100 µg/mL streptomycin) supplemented with 0.26 U/mL Liberase and 166 U/mL DNase I for 45 min at 37 °C with no shaking. The digested tissue was then passed through a cell strainer (70 µm) and washed with cold PBS. Cell counts were determined using the CASY TT cell counter (OLS-Omni Life Science, Bremen, Germany). A total of 10^6^ cells/sample were stained with a Zombie NIR™ Fixable Viability Kit (BioLegend, London, United Kingdom) for 20 min at RT in PBS followed by a 20 min incubation with mouse anti CD16/32 (clone: 93) and stained with appropriate fluorochrome-conjugated antibodies: anti-CD45-PacificBlue (clone: REA737), anti-CD11c-AlexaFluor488 (clone: N418), anti-CD11b-APC (clone: M1/70), anti-CD64-PE/Vio770 (clone: REA286), anti-Ly6G/C-APC/Fire810 (clone: RB6-8C5), anti-CD3-BV510 (clone: 17A2), anti-CD4-PerCP/Cy5.5 (clone: GK1.5), anti-CD8-BrilliantViolet711 (clone: 53-6.7), anti-CD19-SuperBright600 (clone: eBio1D3), anti-SiglecF-PE (clone: E50-2440) for 20 min at 4 °C in FACS Buffer (PBS containing 0.5% FBS). After washing with FACS Buffer, flow cytometric analysis was performed using the Cytek™Aurora, and data were analyzed using FlowJo Software (v10.8.1, Tree Star Inc., San Carlos, CA, USA). Living cells were differentiated by gating to the following populations: Lymphocytes (CD45^+^), B-cells (CD19^+^), T helper cells (CD4^+^CD8^−^, cytotoxic T cells (CD4^−^CD8^+^), dendritic cells (CD11c^+^CD64^−^), interstitial macrophages (CD11b^+^CD11c^+^), and alveolar macrophages (CD11c^+^CD11b^−^SiglecF^+^).

### 2.5. Multiplex Analysis

Soluble receptor concentrations of cytokines (IFN-γ (6.5–4750 pg/mL), TNF-α (15.6–11,400 pg/mL), IL-1β (7.3–5350 pg/mL), IL-12p70 (10.8–7850 pg/mL), IL-4 (6.3–4600 pg/mL), IL-5 (14.1–10,300 pg/mL), IL-13 (17.1–12,500 pg/mL), IL-10 (14.7–10,700 pg/mL)) and chemokines (RANTES (15.4–11,200 pg/mL), CCL3 (2.6–1900 pg/mL), CCL4 (5.4–3950 pg/mL), CCL2 (38.5–28,100 pg/mL), CXCL-1 (8.2–6000 pg/mL), CXCL-2 (3.8–2750 pg/mL), CXCL10 (3.4–2500 pg/mL)) combined in Mix and Match ProcartaPlex-Kits (ThermoFisher Scientific, Dreieich, Germany) of BAL fluid, was analyzed using the multiplexed Luminex xMAP system (Luminex^®^ 100/200™) and the xPONENT^®^ 3.1-Software (Luminex Corp, Austin, TX, USA) according to the manufacturer’s instructions. All measurements were performed in duplicates.

### 2.6. Statistics

Statistical analysis was performed using GraphPad Prism 9.0 (GraphPad Software, La Jolla, CA, USA). After testing the data for normality, normally distributed data were tested for homogeneous variances using Levene’s test and analyzed with an ordinary one-way ANOVA, followed by a Dunnett’s test. Data sets that failed normality tests were compared using the Kruskal–Wallis test followed by a Dunn’s post-hoc test. Values are expressed as means or medians, respectively. For all statistical analyses, *p* values < 0.05 were considered significant. * # *p* < 0.05, ** ## *p* < 0.01, *** ### *p* < 0.001.

## 3. Results

### 3.1. Male or Female Lung Stage Larvae of Schistosoma mansoni Induce Stronger Expression of Inflammation-Associated Genes than Larvae of Both Sexes

**Figure 1:**
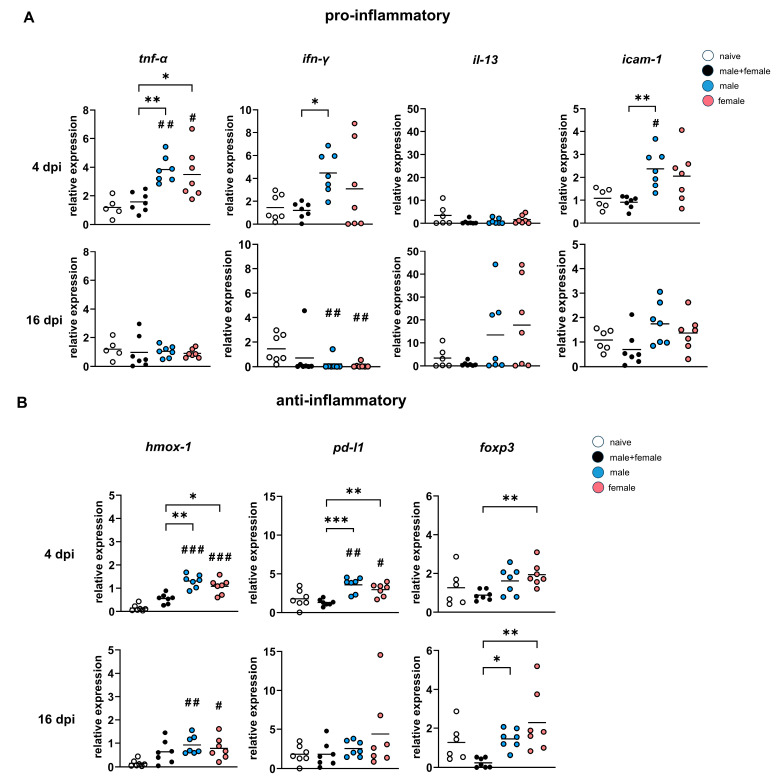
Male or female lung stage larvae of *Schistosoma mansoni* induce stronger regulation of inflammation-associated genes than larvae of both sexes. (**A**) Relative gene expression of pro-inflammatory *tnf-α*, *ifn-γ*, *il-13*, *icam-1*, and (**B**) anti-inflammatory *hmox-1*, *pd-l1*, and *foxp3* in the lungs of *Schistosoma mansoni*-infected mice (male, female, male + female) was analyzed by real-time qPCR 4 and 16 dpi. (*n* = 7, naive control *n* = 6). *p* values resulted either from one-way ANOVA (*tnf-α* 16 dpi, *pd-l1* 4 dpi, *foxp3* 4 dpi) with mean or from Kruskal–Wallis test with median. *p* values < 0.05 were considered statistically significant. * *p* < 0.05, ** *p* < 0.01, *** *p* < 0.001 (mixed-sex vs. single-sex); # *p* < 0.05, ## *p* < 0.01, ### *p* < 0.001 (naive vs. infected).

The time of highest LSL density was determined using mixed cercariae. The peak was reached six days after the infection. Because we cannot exclude differences in the migration speed of unisexual or bisexual cercariae, we chose an early (4 days post-infection, dpi) time point before the peak and a late time point after the peak (16 dpi) for our analyses, since the focus of our analyses was not on a time course but on the differences between unisexual male or female LSL or male and female. Female C57BL/6j mice were infected with 100 male-only, 100 female-only, or a mixture of male and female (100) cercariae for 4 or 16 days. To investigate the immune response to migrating juvenile worms, pro-inflammatory (Figure 1A) as well as anti-inflammatory (Figure 1B) genes were analyzed.

Key pro-inflammatory genes were upregulated 4 dpi in male and female groups, whereby the male + female group was at the same level as the naive control group. The expression of the Th1-associated gene *tumor necrosis factor α* (*tnf-α*) was significantly increased in both single-sex infected groups compared with the male + female group, while the expression levels of *interferon-γ* (*ifn-γ*) and *intercellular adhesion molecule 1* (*icam-1*) were found to be significantly elevated only in the male group. Expression was also increased in the female group, albeit not significantly. At day 16 dpi, pro-inflammatory *ifn-γ* expression was significantly reduced in the male and female groups compared with the naive control group. The *tnf-α* expression levels of the infected groups decreased in comparison to 4 dpi and are at the level of the naive control group. This contrasts with the expression of the Th2-associated gene *interleukin-13* (*il-13*). Gene expression was increased on day 16 p.i., but only in the single-sex infected groups compared with the naive control, albeit not significantly. The expression levels of the anti-inflammatory genes *heme oxygenase-1* (*hmox-1*) and *programmed cell death ligand 1* (*pd-l1*) were significantly higher in the unisexually infected groups compared with the naive control group and the male + female group, with the male-infected group showing the highest value. The female infected group showed a significantly higher *forkhead box p 3* (*foxp3*) relative expression compared with male + female group at day 4 p.i. After 16 days, the relative expression of *foxp3* decreased in the male + female group and was thus significantly lower compared with the male and female groups. Relative expression of *hmox-1* slightly decreased but was still significantly higher than the naive control group. An increase in *pd-l1* expression was shown by the female group at 16 dpi, while that of the male group decreased and was almost at the same level as the male + female and the naive control group. In summary, inflammatory markers are upregulated at the level of gene expression in lung tissue as early as 4 dpi, with the two single-sex infected groups differing significantly from the bisexual control. The single-sex LSL (male or female) induces comparable expression patterns. This affects both Th1 and Th2 responses.

### 3.2. During Lung Migration, Single-Sex Larvae of Schistosoma mansoni Cause a Different Immune Milieu Compared with the Bisexual Control

To analyze the composition of immune cells in the mouse lung during *Schistosoma mansoni* LSL migration, flow cytometric analyses of fresh lung cell suspensions were performed.

**Figure 2:**
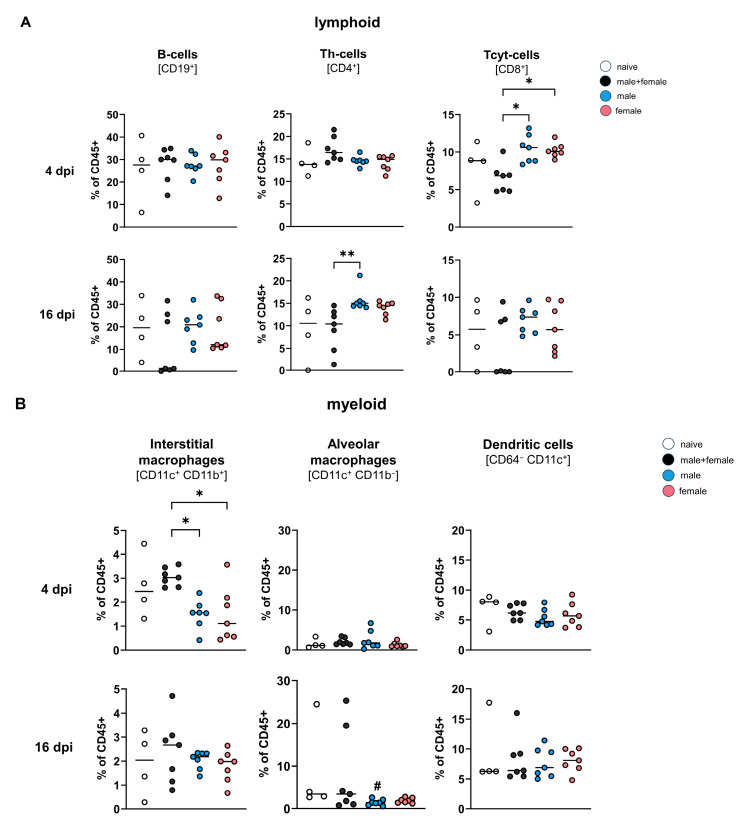
Single-sex lung stage larvae of *Schistosoma mansoni* cause a different immune environment during lung migration than both larval sexes together. (**A**) The percentage of B-cells, T helper cells, cytotoxic T cells, (**B**) interstitial Macrophages, alveolar Macrophages, and Dendritic cells were analyzed by flow cytometry in homogenized lungs from infected (male, female, male + female) and naive mice 4 and 16 dpi (*n* = 7, naive control *n* = 4). *p* values resulted from Kruskal–Wallis test with median. *p* values < 0.05 were considered statistically significant. * *p* < 0.05; ** *p* < 0.01 (mixed-sex vs. single-sex); # *p* < 0.05 (naive vs. infected).

Four days post-infection, significantly higher CD8^+^ cytotoxic T cells were observed in single-sex infected groups compared with the male + female group (Figure 2A; for the gating strategy, see Supplementary Figure S2). In addition, the data showed that there were significantly lower numbers of interstitial macrophages in the lung tissue of the male and female groups compared with the male + female group. After 16 days of infection, CD8^+^ cytotoxic T cells were still higher in single-sex infected groups and interstitial macrophages were lower, but the cell numbers did not reach significance. Furthermore, single-sex infected groups displayed a higher percentage of B-cells at 16 dpi. Significantly more CD4^+^ T helper cells were observed in the male group compared with the male + female group. A significantly lower percentage of alveolar macrophages was observed in the male group than in the naive control group (16 dpi) (Figure 2B). Taken together, these data show that infection with only male or only female cercariae affects the composition of inflammatory cells in the lung during migration in a similar manner. However, the inflammatory response differs markedly from that of a natural infection in both sexes. Of particular note is the significantly increased proportion of CD8^+^ cytotoxic T cells in the single-sex infected groups compared with the bisexual control 4 days after infection. This effect was also maintained 16 days after infection, albeit not significantly.

### 3.3. During Lung Migration of Single-Sex Schistosoma mansoni Larvae, Cytokine Concentrations in the Bronchoalveolar Lavage Are Higher than in Bisexual Infection

**Figure 3:**
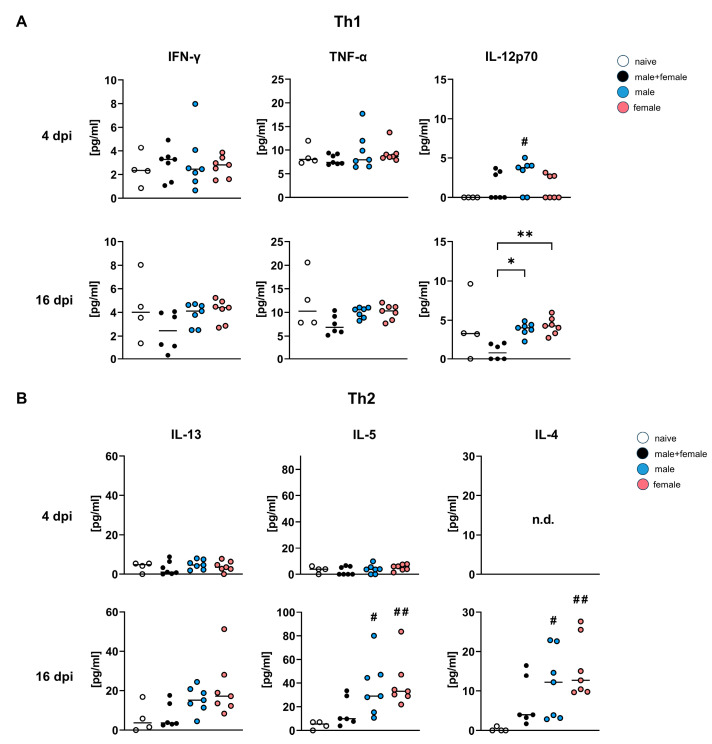
Single-sex *Schistosoma mansoni* infection causes higher cytokine concentrations in the bronchoalveolar lavage compared with bisexual infection. Amounts of (**A**) Th1 and (**B**) Th2 cytokines in BALs of mice infected with *Schistosoma mansoni* (male, female, male + female) for 4 and 16 days and naive control were quantified using multiplex analysis (*n* = 7, naive control *n* = 4). *p* values resulted from Kruskal–Wallis test with median. *p* values < 0.05 were considered statistically significant. * *p* < 0.05, ** *p* < 0.01 (mixed-sex vs. single-sex); # *p* < 0.05, ## *p* < 0.01 (naive vs. infected).

Multiplex analysis was used to determine cytokine (Figure 3A,B, Supplementary Table S1) levels in the bronchoalveolar lavage (BAL) of mice infected with male-only, female-only, or male and female *S. mansoni* cercariae.

Figure 3 illustrates the cytokines that show noticeable differences between male or female and male + female *S. mansoni* infections (for further information on analyzed cytokines and chemokines, see Supplementary Table S1). Cytokine levels of IFN-γ and TNF-α, which are associated with Th1-like inflammation, are almost the same in the infected groups 4 days after infection (Figure 3A). Significantly higher levels of IL-12p70 were observed only in the male-infected group when compared with the naive control. IL-4 was not detected at 4 dpi in any of the groups. At 16 dpi, higher levels of IFN-γ and TNF-α are found in the unisexually infected groups than in the male + female infected group, with a significantly higher level of IL 12p70 in the male-only and female-only infected groups. BAL levels of Th-2-associated cytokines were also higher in the unisexually infected groups than in the male + female infected group, with a significant increase in IL-5 and IL-4 compared with the naive control group (Figure 3B). Overall, there is only a minor difference between groups in cytokine levels at 4 dpi. The differences become more apparent after 16 days of infection. Our results show that cytokine levels in the BAL samples from male or female infected mice do not differ but are substantially higher than those in the BAL from male and female infected mice.

## 4. Discussion

The aim of this study was to analyze the inflammatory milieu in the lung (mouse model) during the passage of *Schistosoma mansoni* larvae, focusing mainly on single-sex infection. We demonstrated that the inflammatory response at the molecular and cellular levels induced by infection with male or female cercariae is quite similar at 4 and 16 dpi. However, we could show that the inflammatory profiles in single-sex infected groups were distinctly different from those of a natural, bisexual infection. At both gene expression and protein levels, a stronger inflammatory response is induced by single-sex infection compared with infection with both parasite sexes. At the cellular level, the significant increase in the percentage of CD8^+^ cytotoxic T cells is evident as early as 4 days after infection. This is an unexpected result since the lung stage larvae are immature and non-mated.

From the skin-penetrating schistosomula to the lung stage, *Schistosoma* spp. larvae undergo extensive morphological and biochemical adaptations that are primarily due to the conversion of an aquatic free-living organism to a body-dwelling endogenous parasite stage. In particular, the adaptation or stripping of the outer layer of the skin (glycocalyx) schistosomula towards the development of a tegument leads to the emergence of a large number of new antigens that can stimulate immune responses very efficiently [28]. As early as 1992, Dean & Mangold postulated that the lung represents a principal site of worm elimination [29]. This is supported by studies with radiation-attenuated cercariae, which show that the reduced migration speed of the irradiated cercariae combined with an augmentation of the immunogenicity of the larval stage allows the host to develop a more robust immune response [30]. In reinfection studies, the radiation-attenuated vaccine against schistosome larvae (RA) has been shown to confer resistance to reinfection in mice by stopping migration through immune cell clusters in the lungs. In addition, these studies showed a consistently high protective effect due to a balanced Th1/Th2 response [13,31,32,33,34].

When analyzing T-cell responses with FACS 7-, 14-, and 21 dpi in the thoracic (lung-draining) lymph nodes during bisexual schistosomula lung migration, a noteworthy rise of Th2 cell IL-4 expression was observed, but no increase of Th1 IFN-γ [35], which goes in line with the multiplex data shown in the presented study (Figure 3A). As infection progresses, an IL-4 increase can be further observed on SWAP-stimulated spleen and liver cells from 4-week-old infected mice [36]. In our study, single-sex infected groups showed even higher IL-4, IL-5, and Il-13 levels compared with the bisexual control after 16 dpi in the BAL. However, the gene expression data showed significantly higher levels of *ifn-γ* and *tnf-α* in single-sex infected groups at 4 dpi. Houlder et al. implicated a mixed Th1/Th2 response as a hallmark of pre-patent infection in mice naturally infected with both sexes [37]. Our data indicate an emerging Th2 response at the gene and protein level as early as 16 dpi, while in the male + female group, no polarization is identifiable at this time.

However, the increased gene expression of *tnf-α* and *ifn-γ* could not be verified at the protein level. The gene expression was determined in the lung tissue with, although reaching significance, only a minor effect size. Therefore, it is obvious that the effect of gene expression cannot be mapped to the protein level in the BAL. In order to investigate the precise inflammatory process, further time points between 4 and 16 dpi need to be examined.

Up-regulation of *hmox-1*, which is known to be an important regulator of inflammation in asthma and hypoxic lung injury [38] as well as crucial for transition from inflammation to wound healing responses [39], was significantly higher in single-sex infected mice compared with the naturally infected group as early as 4 days after infection (Figure 1A,B). During *S. japonicum* LSL migration, increased *hmox-1* gene expression is already seen as early as 3 dpi, which might be attributed to the tissue damage associated with hemorrhage caused by migrating larvae [40]. Accordingly, the significantly higher *hmox-1* expression in single-sex infected groups in this study might be linked to more severe tissue damage caused by single-sexual migrating *S. mansoni* LSL. This assumption is supported by the enhanced expression of *icam-1* in the single-sex infected groups after only 4 days of infection, since ICAM-1, as an endothelial activation marker, can also be associated with the wound healing process [41].

In addition, our data indicate significantly increased expression of *pd-l1* in single-sex infected groups, peaking in the single-sex male group. It has been previously described that *S. mansoni* is able to upregulate PD-L1 on macrophages to suppress the host immune system in bisexual infection models [42]. We have not observed a decrease in T cells, but a significantly higher percentage of CD8^+^ cytotoxic T cells in both single-sex infected groups (4 dpi) and a significantly higher cell count of CD4^+^ T helper cells (16 dpi) (Figure 2A). There are conflicting studies on the ability of PD-L1 to either induce T-cell anergy [42,43] or co-stimulate T-cell proliferation through the PD-L1-PD-1 interaction [44]. A 1979 study showed that cytotoxic T cells directed against alloantigens have a marked affinity for schistosome larvae carrying passively acquired host-derived alloantigens [45]. This might explain the increase in CD8^+^ cytotoxic T cells at 4 dpi measured in the present study.

Activation and proliferation of Foxp3^+^Treg cells play an important role in immune suppression and, thus, parasite survival. This has been previously demonstrated in infections with filariae [46,47,48] and intestinal nematodes [49,50,51]. However, an expansion of Foxp3^+^Treg cells could not be confirmed for an early bisexual *Schistosoma mansoni* infection [35]. In the present study, we have shown a significant upregulation of *foxp3* gene expression, exclusively in the male or female infected groups, suggesting that male and female larvae promote their own survival by hijacking the host immune response.

The results of the present study provide the first indications that divergent immune responses induced by the individual sexes of schistosomes, or by both, become apparent early in the lung stage of the parasites. Based on our own preliminary data and the data of others [20,24], we assumed that male and female schistosomes induce different inflammatory profiles, with a greater immunomodulatory effect due to the pre-infection with male cercariae. This is especially relevant as we demonstrated that animals previously infected with male cercariae and re-infected within 6 weeks showed a significant reduction in worm burden, while mice previously infected with female cercariae showed a significant reduction in hepatic fibrosis 8 weeks after re-infection. The reduction of worm burden following re-infection was about 33 to 34% in the group initially infected with male cercaria only compared with the female group. However, we also found that this reduction in the rate of new infections depends on a short period of time between the first and second infections [25,26]. We discussed the inflammatory environment in the lung caused by the first single-sex infection as a possible cause for the reduction of the worm burden. A first infection with female cercariae had no effect on the worm burden. Furthermore, we and others have shown that a single-sex infection triggers a non-polarized Th1/Th2 immune response [52,53]. This became particularly clear in transcriptome analyses of spleen tissue from mice infected with male or female or with male and female cercariae 8 weeks after infection. A clear clustering of the regulated genes in the three different experimental groups became apparent. The number of regulated genes in the spleens of mice infected with male cercariae (1293) exceeded that of the female group (512) by a factor of two. The limitations of the present studies lie in the confined representation of the inflammatory process over time, as we have not shown a detailed inflammatory process. The assumed time points of larval entry and the end of larval migration are presented. The transient inflammatory events in between and especially afterwards will have to be part of further analyses. This includes in-depth molecular and immunological analyses in order to draw a more detailed immune landscape in the lungs after unisexual or bisexual infection, especially following re-infection. Nevertheless, we were able to show for the first time that at the time points studied, male or female larvae induce a comparable immune environment, while male and female larvae clearly differ from the single-sex infection.

## 5. Conclusions

Interest in schistosomiasis research is undoubtedly due to ongoing pressure for the development of a vaccine against the multicellular parasite *Schistosoma* spp., thus reducing reinfection rates in endemic areas and limiting transmission. Since it was postulated that the lung represents a principal site of worm elimination, this study focused on the inflammatory milieu triggered by male or female schistosomes during lung passage. This study is the first to demonstrate that the immune response during the early lung phase of a single-sex infection with *Schistosoma mansoni* differs significantly from the natural, asexual infection. The differences are clearly evident at the level of gene expression, with corresponding manifestations in the cellular composition of the inflammatory lung cell infiltrate and the concentrations of cytokines in the bronchoalveolar lavage (BAL). Regardless of how long a particular immune milieu can be maintained in the lungs after the migration of juvenile schistosomes and thus eliminate subsequent schistosomes, with regard to the identification of therapy targets to achieve resistance to re-infection, it is of great scientific interest to further analyze the differences in the inflammatory potential of male and female parasites, especially after reinfection.

## Data Availability

The original contributions presented in the study are included in the article/supplementary material; further inquiries can be directed to the corresponding author.

## Supplementary Materials

The following supporting information can be downloaded at: https://www.mdpi.com/article/10.3390/pathogens12121432/s1, Figure S1: Migration Peak, Figure S2: Gating Strategy; Table S1: Multiplex analysis.
