# Supplementary material for: Individually or as a Team—The Immunological Milieu in the Lung Caused by Migrating Single-Sex or Mixed-Sex Larvae of Schistosoma mansoni
Source: Pathogens. 2023 Dec 8;12(12):1432. doi: 10.3390/pathogens12121432 (PMC10746046; doi:10.3390/pathogens12121432)
Supplement: Supplementary file 1 [file pathogens-12-01432-s001.zip › pathogens-2735491-supplementary.pdf]

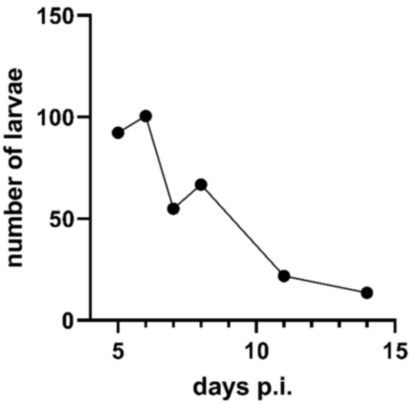

Supplementary Figure 1. Number of *Schistosoma mansoni* larvae counted in the mouse lung with peak on day 6 after infection. The mice were infected with 300 male and female cercariae. The data are expressed as mean values, with n=5.

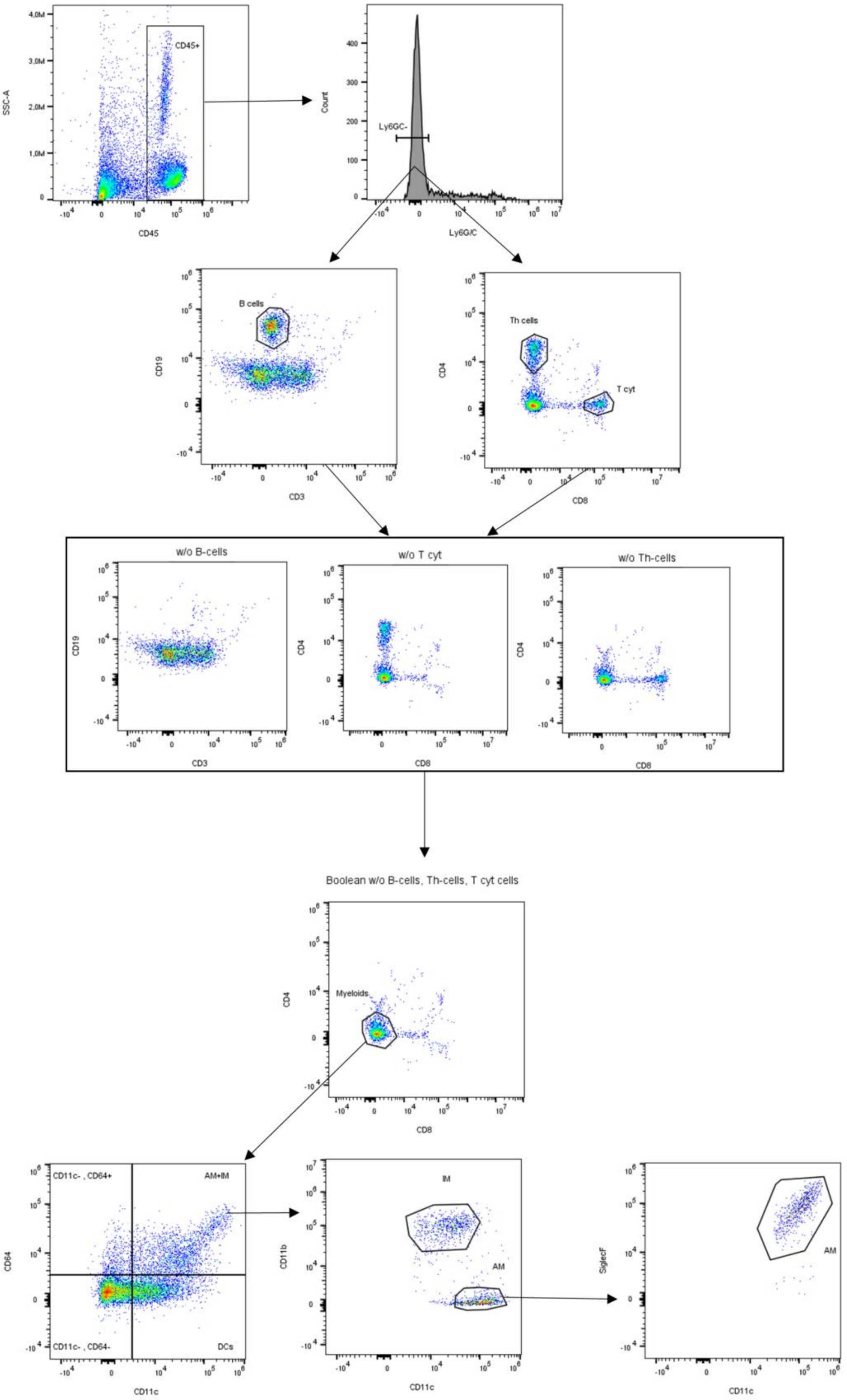

Supplementary Figure 2. Gating strategy of lymphoid and myeloid lung cells.T cyt: cytotoxic T cells; Th-cells: T helper cells; Myeloids: myeloid cells; AM: alveolar macrophages; IM: interstitial macrophages; DCs: dendritic cells.

Supplementary Table 1. Cytokines and chemokines of female only, male only or male and female *Schistosoma mansoni* infected mice measured in BAL fluids with a Multiplex assay. The data are expressed as mean values, with n=7 for the infected groups and n=6 for the naive control, measured in (pg/ml).

	4 days p.i.				16 days p.i.			
[pg/ml]	**female**	**male**	**male+female**	**naive**	**female**	**male**	**male+female**	**naive**
IL10	6,63	9,01	3,60	9,73	8,29	3,70	0,00	14,07
IL-1β	0,00	0,00	0,12	0,16	0,00	0,86	0,78	0,14
CXCL1	7,28	8,61	5,75	6,70	17,11	20,51	19,99	10,57
CXCL2	4,88	5,77	6,07	4,72	6,44	5,94	3,33	7,11
CXCL10	9,08	10,32	8,32	10,15	53,08	55,55	31,66	10,37
CCL2	38,74	40,41	42,09	55,23	53,99	49,42	84,63	43,54
CCL3	0,30	0,56	0,43	0,16	1,86	2,06	0,93	0,80
CCL4	1,50	1,83	1,56	1,76	4,05	4,46	2,77	2,46
RANTES	0,84	0,92	2,28	3,61	10,46	10,88	11,24	5,58
